# LimROTS: a hybrid method integrating empirical Bayes and reproducibility-optimized statistics for robust differential expression analysis

**DOI:** 10.1093/bioinformatics/btaf570

**Published:** 2025-10-11

**Authors:** Ali Mostafa Anwar, Akewak Jeba, Leo Lahti, Eleanor Coffey

**Affiliations:** Turku Bioscience Centre, University of Turku and Åbo Akademi University, Turku 20520, Finland; Department of Computing, Faculty of Technology, University of Turku, Turku 20014, Finland; Department of Computing, Faculty of Technology, University of Turku, Turku 20014, Finland; Turku Bioscience Centre, University of Turku and Åbo Akademi University, Turku 20520, Finland

## Abstract

**Motivation:**

Differential expression analysis plays a vital role in omics research enabling precise identification of features that associate with different phenotypes. This process is critical for uncovering biological differences between conditions, such as disease versus healthy states. In proteomics, several statistical methods have been used, ranging from simple t-tests to more advanced methods like DEqMS, limma and ROTS. However, a flexible method for reproducibility-optimized statistics tailored for clinical omics data has been lacking.

**Results:**

In this study, we developed LimROTS, a hybrid method that integrates a linear regression model and the empirical Bayes approach with reproducibility optimized statistics, to create a novel moderated ranking statistic, for robust and flexible analysis of proteomics data. We validated its performance using twenty-one proteomics gold standard spike-in datasets with different protein mixtures, MS instruments, and techniques for benchmarking. This hybrid approach improves accuracy and reproducibility of complex proteomics data, making LimROTS a powerful tool for high-dimensional omics data analysis.

**Availability and implementation:**

LimROTS has been implemented as an R/Bioconductor package, available at https://doi.org/doi:10.18129/B9.bioc.LimROTS. Additionally, the code used in this study is available in GitHub repository https://github.com/AliYoussef96/LimROTSmanuscript.

## Graphical abstract

**Figure:**
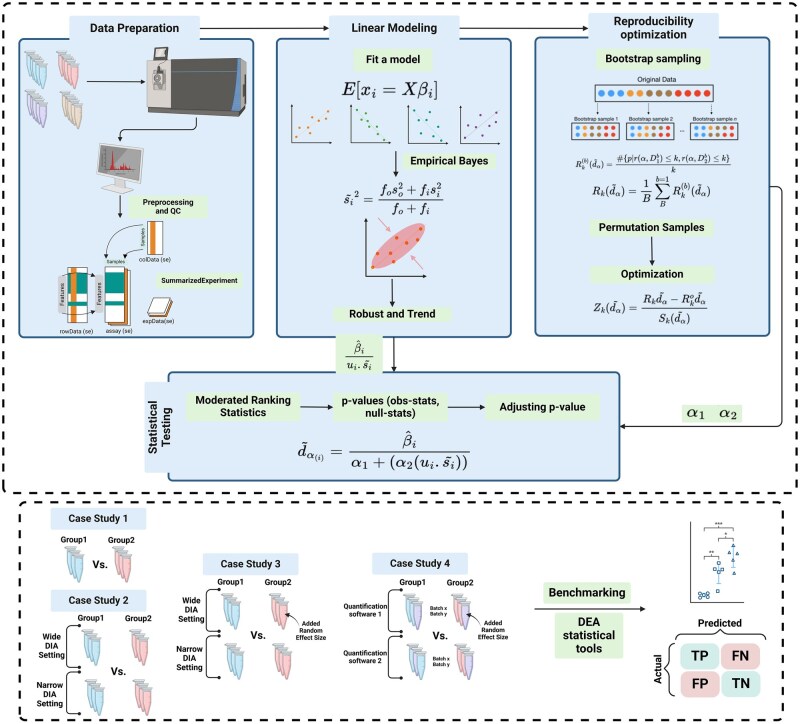
The graphical abstract presented in this study was created in BioRender. Youssef, A. (2025) https://BioRender.com/hl9oebo

## 1 Introduction

Advances in mass spectrometry technologies and data processing methods have expanded the depth of proteomic detail attainable from biospecimens. These large datasets encode complex biological information that can help to advance precision medicine (Deng *et al.* 2025, He *et al.* 2025). The ability to detect phenotype-specific protein changes not only enhances our understanding of underlying biological mechanisms, it also holds promise for the discovery of novel biomarkers for early disease diagnosis, prognosis, and monitoring. Moreover, it aids the identification of potential drug targets for the development of new therapeutic strategies (Niu *et al.* 2022, Meissner *et al.* 2022).

One statistical tool that has recently gained attention for its high performance in proteomics data analysis is ROTS (Reproducibility-Optimized Test Statistic). ROTS is a statistical method specifically designed for differential expression analysis (DEA) of microarray RNA data, including applications in proteomics (Elo *et al.* 2008, 2009, Suomi *et al.* 2017, Dowell *et al.* 2021, Peng *et al.* 2024). It identifies differentially expressed proteins (DEPs) by optimizing the test statistic based on reproducibility across bootstrap datasets. Instead of relying on traditional fixed test statistics, ROTS searches for the optimal combination of statistical parameters that maximize the reproducibility of significant results. This data-driven approach improves the detection of true biological signals while controlling for false discoveries (Elo *et al*. 2008, Suomi *et al.* 2017), making ROTS a robust tool for analyzing proteomics datasets.

A highly utilized tool that has been developed for microarray and RNA-Seq data is limma (Ritchie *et al.* 2015). It applies linear models to the data which help to consider multiple variables and complex experimental designs. limma applies empirical Bayes methods to improve statistical power and control for false discoveries, making it particularly effective for high-dimensional data like proteomics (van Ooijen *et al.* 2018, Peng *et al.* 2024). By combining robust statistical techniques with flexible model designs, limma has become a cornerstone tool for identifying DEPs in a variety of biological contexts (van Ooijen *et al.* 2018).

Although ROTS has shown superior performance in proteomics benchmarking studies (Dowell *et al.* 2021, Peng *et al.* 2024), its indicated version (Elo *et al.* 2008, 2009, Suomi *et al.* 2017) lacks flexibility for complex clinical proteomics datasets, as it does not account for covariance in the data. For example, the effect of covariates such as age and gender on the data outcome, and technical variations such as batch effects, need to be taken into consideration by accounting for these variations (Yan *et al.* 2024, Loo *et al.* 2024). Omics studies involving DEA have shown that the empirical Bayes method provides a robust approach when implemented within the limma framework (Ritchie *et al.* 2015). In this study, we developed LimROTS, a hybrid method integrating the linear model and the empirical Bayes method from the limma framework with the Reproducibility-Optimized Statistics from ROTS, by introducing a new moderated ranking statistic, for robust and flexible analysis of proteomics data. To test LimROTS, 21 gold standard spike-in datasets (Gotti *et al.* 2021, 2022, Peng *et al.* 2024) were utilized and compared to limma, ROTS, MSstats, DEqMS, DEP, ANOVA, t-test, and SAM. Furthermore, we assessed LimROTS, limma, and ROTS in a real-world clinical dataset using Alzheimer’s disease (AD) samples from the University of Pennsylvania School of Medicine Brain Bank and the Baltimore Coroner’s Office (Johnson *et al.* 2020).

## 2 Materials and methods

### 2.1 Integrating linear regression model and empirical Bayes with reproducibility-optimized statistics

LimROTS integrates the statistical principles of linear regression model and empirical Bayes as implemented in limma (Ritchie *et al.* 2015) and extends that as an optimization problem, solved by the reproducibility optimization statistics (Suomi *et al.* 2017). Therefore, in principle, LimROTS inherits part of the mathematical rationale from both methods, with a new moderated ranking statistic.

The new moderated ranking statistic ${\tilde{d}}_{\alpha_{\left(i\right)}}$ introduced in LimROTS can be represented as:


(1) $${\tilde{d}}_{\alpha_{\left(i\right)}}=\frac{{\hat{\beta }}_{i}}{\alpha_{1}+(\alpha_{2}(u.\;{\tilde{s}}_{i}\;))},$$

where ${\hat{\beta }}_{i}$ is the effect (coefficient) due to the experiment conditions, $\alpha_{1}$ and $\alpha_{2}$ are reproducibility optimized parameters, u is the un-scaled standard deviation, and ${\tilde{s}}_{i}$ is the posterior residual standard deviation.

To calculate ${\tilde{d}}_{\alpha_{\left(i\right)}}$, first, a feature-wise linear model is fitted, which allows for the handling of complex experimental design, this can be represented as:


(2) $$E[x_{i}]=X{\hat{\beta }}_{i},$$

where $x_{i}$ is the observed expression value of a feature, X is the design matrix of an experiment, and coefficient ${\hat{\beta }}_{i}$ being estimated by the linear model, represents how much the expression changes due to the experimental conditions (effect size). In this step, covariates can be incorporated through the design matrix (X). This allows LimROTS to adjust for potential confounders such as batch effects, injection order, or demographic factors, enabling the computation of moderated statistics that reflect the condition effect while accounting for other sources of variation.

The unscaled standard deviation in Equation (1) can then be calculated as:


(3) $$u=\sqrt{c^{T}{\left(X^{T}X\right)}^{-1}c},$$

where *c* is the contrast vector for the coefficient being tested, and $c^{T}$is its transpose. Using parametric empirical Bayes technique to borrow information between features in a dynamic way, the ${\tilde{s}}_{i}^{2}\;$which is the posterior residual variance is used to adjust the variance to account for uncertainty in the features measurements and combines it with prior information estimated from the data, leading to empirical Bayes variance shrinkage, which can be calculated as;


(4) $${\tilde{s}}_{i}^{2}=\frac{f_{o}s_{o}^{2}+f_{i}s_{i}^{2}}{f_{o}+f_{i}},$$

where $f_{o}$ is the prior degree of freedom (a global parameter) calculated from the whole dataset, $s_{o}^{2}$ is the prior variance (a global parameter) calculated from the whole dataset, $f_{i}$ is the residual degree of freedom calculated for each feature (a feature specific parameter), and $s_{i}^{2}$ is the observed residual variance for feature i (a feature specific parameter). The prior degree of freedom $f_{o}$ and the prior variance $s_{o}^{2}$ are estimated using squeezeVar function in limma R package. This function implements an empirical Bayes algorithm proposed by Smyth (2004).

Equation (4) allows a more reliable estimate of variance for each feature, as well as more statistical confidence in the estimates, because the final variance estimate for each feature (${\tilde{s}}_{i}^{2}$) is a balance between two things: the feature specific variance, and the global variance across all the features in the experiment. In other words, each feature specific variance is shrinkage to a common variance estimated from all the features in the dataset.

The optimization parameters $\alpha_{1}$ and $\alpha_{2}$ are estimated by maximizing the reproducibility by z-type statistics $Z_{k}({\tilde{d}}_{\alpha })$. To calculate the $Z_{k}({\tilde{d}}_{\alpha })$, the average reproducibility score $R_{k}({\tilde{d}}_{\alpha })$ has to be calculated from bootstrap datasets as:


(5) $$R_{k}({\tilde{d}}_{\alpha })=\frac{1}{B}\sum_{b=1}^{B}R_{k}^{\left(b\right)}({\tilde{d}}_{\alpha }),$$

where for each pair of bootstrap datasets $D_{1}^{\left(b\right)}$ and $D_{2}^{\left(b\right)}$ the reproducibility $R_{k}^{\left(b\right)}({\tilde{d}}_{\alpha })$ can be computed as:


(6) $$R_{k}^{\left(b\right)}({\tilde{d}}_{\alpha })=\frac{\#\{i|r(\alpha ,D_{1}^{b})\leq k,\;r(\alpha ,D_{2}^{b})\leq k\}}{k},$$

where {i|…} represents the set of features that satisfy the condition of being less than or equal to k (k equal to cutoff of considering a feature as a top rank feature) in both $D_{1}^{\left(b\right)}$ and $D_{2}^{\left(b\right)}$, $r(\alpha ,D_{1}^{b})$ is the rank of feature p in the dataset $D_{1}^{\left(b\right)}$ with respect to the statistic ${\tilde{d}}_{\alpha }$ and the same for $r(\alpha ,D_{2}^{b})$, then scaled by the k.

Moreover, the $Z_{k}({\tilde{d}}_{\alpha })$ can be estimated by:


(7) $$Z_{k}({\tilde{d}}_{\alpha })=\frac{R_{k}{(\tilde{d}}_{\alpha })-R_{k}^{o}{(\tilde{d}}_{\alpha })}{S_{k}({\tilde{d}}_{\alpha })}.$$

The standard deviation $S_{k}({\tilde{d}}_{\alpha })$ can be computed by square root the variance, calculated as;


(8) $$S_{k}^{2}=\frac{\sum_{b=1}^{B}{\left[R_{k}^{\left(b\right)}({\tilde{d}}_{\alpha })-R_{k}({\tilde{d}}_{\alpha })\right]}^{2}}{B-1},$$

where B is the number of bootstrapping datasets. The $R_{k}^{o}({\tilde{d}}_{\alpha })$ is the null reproducibility estimate of ${\tilde{d}}_{\alpha }$ in random (permutated) data. Finally, to avoid the need of pre-specified k, LimROTS maximize the $Z_{k}({\tilde{d}}_{\alpha })$ over a lattice of (α,k)-pairs.

We use 1000 bootstrap iterations for both ROTS and LimROTS throughout this study. This is the recommended number of bootstraps for ROTS (Suomi *et al.* 2017). Finally, LimROTS evaluates statistical significance using a non-parametric approach by building an empirical null distribution from the test statistic defined in Equation (1), calculated under sample label permutations. Specifically, the empPvals() function from the qvalue package (Storey 2002,   2011) generates empirical *P*-values by comparing the observed statistic to a pooled distribution of permuted statistics across all features.

### 2.2 Differential expression analysis methods used for benchmarking

In this study, LimROTS was compared with several statistical methods that are widely used in proteomics studies (MSstats, DEqMS, DEP, limma, ROTS, SAM, t-test, and ANOVA; for the Bioconductor packages, we used Bioconductor version 3.20). MSstats applies a fixed or mixed effects model, depending on the experimental design, to detect proteins with differential abundance (Choi *et al.* 2014). DEqMS is a limma-based method for analyzing differential protein expression in mass spectrometry data. It accounts for the correlation between protein variance and the number of PSMs or peptides used, leading to more accurate estimates of protein variability (Zhu *et al.* 2020). DEB performs differential expression analysis using protein-specific linear models combined with empirical Bayes methods from the limma framework. It then calculates the local false discovery rates (FDRs) using the fdrtool package (Zhang *et al.* 2018). The statistical method ANOVA is used to determine whether differences in group means are statistically significant or due to random variation (Kerr *et al.* 2000). Limma uses linear models to evaluate differential expressions, supplemented by empirical Bayes statistics for result refinement (Ritchie *et al.* 2015). ROTS adjusts a t-statistic to correspond with the inherent properties of the data, providing a ranking of features according to their statistical importance in demonstrating differential expressions between two or more groups (Suomi *et al.* 2017). SAM identifies proteins with differential expression by assigning a score based on the difference in group means relative to the standard deviation and assesses significance through permutation testing (Tusher *et al.* 2001). The Student’s t-test is a statistical method used to assess whether the means of two groups differ significantly.

### 2.3 Datasets used for method benchmarking

Table 1 shows twenty gold standard spike-in datasets adapted from (Peng *et al.* 2024) including 12 label-free data-dependent acquisition (DDA) datasets and 7 data-independent acquisition (DIA). These datasets were used to evaluate the performance of LimROTS in comparison to other statistical methods. The preprocessing steps applied to each dataset were based on the benchmarking study by Peng *et al.* (2024). For all the methods used in this study (with the exception of MSstats), all datasets were quantified using directLFQ (Ammar *et al.* 2023). In brief, directLFQ gathers peptide intensities, performs protein-level normalization across all samples, integrates the signals to estimate protein abundance, and accounts for missing data to yield a consistent protein matrix. We then imputed missing values, without any additional global normalization (i.e. no median or quantile normalization). In MSstats, global normalization was omitted, and Tukey’s median polish was used as the summarization method.

**Table 1.** Datasets used for workflow benchmarking.^a^

Dataset	ID	Technique	Mixture	Instrument	Samples/conditions	Features	Used in case study
HYEtims735	PXD028735 (Van Puyvelde *et al.* 2022)	DIA	Human+yeast+*E. coli*	TimsToFpro	18/2	11 310	1
MYtims709	PXD034709 (Lou *et al.* 2023)	DIA	Mouse + yeast	TimsTOF Pro	35/6	10 912	1
HEof_n600	PXD026600 (Gotti *et al.* 2021, 2022)	DIA	UPS1 + E. coli	Orbitrap Fusion ETD	24/8	2189	1, 2, 3, and 4
HEof_w600	PXD026600 (Gotti *et al.* 2021, 2022)	DIA	UPS1 + E. coli	Orbitrap Fusion ETD	24/8	2018	1, 2, 3, and 4
HYtims134	PXD036134 (Koopmans *et al.* 2023)	DIA	human + yeast	TimsToF pro	9/3	6574	1
HEqe777	PXD019777 (Kalxdorf *et al.* 2021)	DIA	Human + E. coli	Q Exactive HF	9/3	7016	1
HEqe408	PXD018408 (Dowell *et al.* 2021)	DIA	Human + E. coli	Q Exactive	16/2	4860	1
HYE5600735	PXD028735 (Van Puyvelde *et al.* 2022)	DDA	Human+yeast + *E. coli*	SCIEX Triple TOF5600	18/2	2880	1
HYE6600735	PXD028735 (Van Puyvelde *et al.* 2022)	DDA	Human+yeast+ *E. coli*	SCIEX Triple TOF6600	18/2	3522	1
HYEqe735	PXD028735 (Van Puyvelde *et al.* 2022)	DDA	Human+yeast+*E. coli*	Orbitrap QE-HFX	24/2	5960	1
HYEtims735	PXD028735 (Van Puyvelde *et al.* 2022)	DDA	Human+yeast+*E. coli*	TimsToF pro	14/2	5907	1
HYtims134	PXD036134 (Koopmans *et al.* 2023)	DDA	Human+yeast	TimsToF pro	9/3	2503	1
HEtims425	PXD021425 (Kalxdorf *et al.* 2021)	DDA	Human+*E. coli*	TimsToF pro	9/3	5200	1
YUltq006	PDC000006 (Paulovich *et al.* 2010)	DDA	Yeast+UPS1	LTQ-Orbitrap	15/5	1198	1
YUltq099	PXD002099 (Pursiheimo *et al.* 2015)	DDA	Yeast+UPS1	LTQ Orbitrap Velos	15/5	1515	1
YUltq819	PXD001819 (Ramus *et al.* 2016)	DDA	Yeast+UPS1	LTQ Orbitrap Velos	27/9	1067	1
HEqe408	PXD018408 (Dowell *et al.* 2021)	DDA	Human+*E. coli*	Q Exactive	16/2	2960	1
HYqfl683	PXD007683 (O’Connell *et al.* 2018)	DDA	Human+yeast	Orbitrap Fusion Lumos	11/3	7160	1
HYEtims777	PXD014777 (Prianichnikov *et al.* 2020)	DDA	Human+yeast+*E. coli*	TimsToF pro	6/2	5965	1

a The acronyms indicating the datasets names (first column), incorporate the mixture type, mass spectrometry instrument, and the final three digits of the dataset ID (for instance, HYE5600735, where H represents Human, Y denotes Yeast, and E symbolizes E. Coli). The “5600” as SCIEX Triple TOF 5600 MS instrument was utilized, and 735 from PXD028735.

For DIA quantification, Spectronaut 18 (Bruderer *et al.* 2017) and DIA-NN v1.8.1 (Demichev *et al.* 2020) were used, with directLFQ (Ammar *et al.* 2023) as expression matrix with no further global normalization. Missing values were imputed using the sequential imputation method via the impSeq function in R (Verboven *et al.* 2007) for Spectronaut, and MinDet for DIA-NN (Lazar *et al.* 2016) selected based on prior benchmarking results (Wang *et al.* 2020, Peng *et al.* 2024). The impSeq approach systematically imputes missing values in an incomplete observation by minimizing the determinant of the covariance in the augmented data matrix. Subsequently, the observation is integrated into the comprehensive data matrix, and the algorithm advances to the subsequent observation with missing values. MinDet performs a left-censored imputation using a deterministic minimal value approach for the missing values.

For DDA, MaxQuant v2.1.0.0 (Prianichnikov *et al.* 2020) and FragPipe v20.0 (Kong *et al.* 2017) were used with directLFQ as expression matrix, and missing values were imputed by sequential imputation using impSeq and seqKNNimp (Kim *et al.* 2004) functions. seqKNNimp divides the dataset into an incomplete subset containing missing values and a complete subset without any missing values. A missing value is imputed using the weighted mean of the relevant column from the nearest neighbouring units within the whole dataset. Upon the imputation of all missing values for a specific unit, the unit is transferred to the full dataset and utilized for the imputation of the remaining units in the incomplete dataset.

Sample size ranged from 6 to 35 with conditions from 2 to 9, which means a dataset may have more than one contrast. Therefore, all the contrasts for datasets have been considered in the comparison. The quantified features in these datasets extended from 1067 to 11 310.

We evaluated the performance of LimROTS compared to the previously mentioned statistical methods across four case studies of increasing experimental complexity. In Case Study 1, all datasets were processed using directLFQ (also in other case studies), which performs protein summarization, quantification, and internal normalization, followed by imputation and with no further global normalization. All possible contrasts were analyzed for each dataset, averaging two contrasts per dataset, resulting in approximately 66 comparisons in the 7 DIA datasets and 71 in the 12 DDA datasets (Table 1). While in Case Study 2 (with same preprocessing steps as in case study 1), we assessed method performance in the presence of batch effects. To simulate these effects, we combined two UPS1 + E. coli DIA datasets (HEof_w600 and HEof_n600), which contain identical samples processed with the same software but quantified using different isolation window settings (wide and narrow). These settings were treated as distinct batches for analysis.

Case Study 3 (with same preprocessing steps as in case study 1) simulated a stronger batch effect. As in Case Study 2, the two UPS1 + E. coli DIA datasets were merged. However, to simulate a more pronounced batch effect, we introduced an artificial signal by randomly selecting 500 E. coli proteins, expected to show no variation between contrasts. Then we added a random shift ranging from 5 to 20 to their log2-intensity values, but only in the wide-window samples. This manipulation was designed to create a technical variation in the wide DIA samples. In Case Study 4, we used a dataset from (Gotti *et al.* 2021, 2022) to evaluate method performance under a substantially stronger batch effect compared to Case Studies 2 and 3. We merged identical UPS1 + E. coli DIA samples quantified using two completely different software tools, Spectronaut and ScaffoldDIA. Samples with varying UPS1 spike-in concentrations (ranging from 0.1 to 50 fmol per microgram of E. coli proteins) were grouped into two categories: low UPS1 concentration (0.1 to 2.5 fmol) and high UPS1 concentration (5 to 50 fmol), rather than treating each concentration separately. To further increase the batch effect, we assigned two artificial batch labels to the groups with an unbalanced sample ratio. Additionally, we added an effect size of 10 to the log2-intensity values of 100 randomly selected E. coli proteins in all samples belonging to one of the artificial batches.

The UPenn cohort data (Dammer *et al.* 2019, Johnson *et al.* 2020) were downloaded from Synapse (ID: syn20933797, syn21441786) and used in this study as an example of a real-world clinical dataset. Samples were analysed on a Q-Exactive Plus mass spectrometer essentially as described in (Seyfried *et al.* 2017). Then, using the MaxQuant protein group results, we set the razor unique peptide number to be two or more. Protein groups that showed more than 50% of missing values were removed, impSeq function was used for imputing the rest of the missing values, and ComBat from the SVA package (Leek *et al.* 2012) was used to correct for the batch. Finally, we performed DEA between the Alzheimer’s disease and the Control group, using LimROTS, limma and ROTS.

### 2.4 Significant cutoffs and performance evaluation metrics

In this study, proteins with an adjusted *P*-value <0.05 were identified as significant proteins. Five evaluation metrics (Normalized Matthews correlation coefficient, F1 score, G-mean, Balanced accuracy, and pAUC) were used to evaluate the methods performance. Normalized Matthews correlation coefficient (nMCC) assesses the quality of binary classifications by considering all components of the confusion matrix and is robust to class imbalance. It is calculated as follows:


$$\mathrm{nMCC}=\frac{1}{2}(\frac{TP\times TN-FP\times FN}{\sqrt{\left(TP+FP)(TP+FN)(TN+FP)(TN+FN\right)}}+1).$$

The harmonic mean of precision and recall (F1 score) is particularly useful when false positives and false negatives are equally important. It can be computed as:


$$F1=\frac{2\times \text{Precision}\times \text{Recall}}{\text{Precision}+\text{Recall}}=\frac{2TP}{2TP+FP+FN}.$$

The geometric mean of sensitivity and specificity (G-mean)reflects the balance between sensitivity and specificity, making it suitable for evaluating classifiers on imbalanced data. It can be calculated as:


$$G-\text{mean}=\sqrt{\text{Sensitivity}\times \text{Specificity}}=\sqrt{\frac{TP}{TP+FN}\times \frac{TN}{TN+FP}}.$$

Balanced accuracy is the average of sensitivity and specificity:


$$\text{Balanced}\;\text{Accuracy}=\frac{1}{2}(\frac{TP}{TP+FN}+\frac{TN}{TN+FP}).$$

The partial area under the ROC curve (pAUC) represents the area under the curve up to a specified false positive rate (FPR) threshold (e.g. 0.05), emphasizing performance in the low-FPR region. This focus is especially advantageous in biomedical applications where minimizing false positives is critical.

Finally, we aggregate the true positives (TP), true negatives (TN), false positives (FP), false negatives (FN), and *P*-values for all comparisons by dataset and software. Using this information, we applied the PRROC tool in R (Grau *et al.* 2015) to construct precision-recall curves (PR-curves).

All the evaluation metrics ranges from 0 (random prediction) to 1 (perfect prediction). For each benchmarked method, the median of each metric was computed across datasets within each case study to summarize overall performance.

## 3 Results

### 3.1 Case study 1: Evaluating LimROTS performance on gold standard spike-in datasets

**Figure 1.**
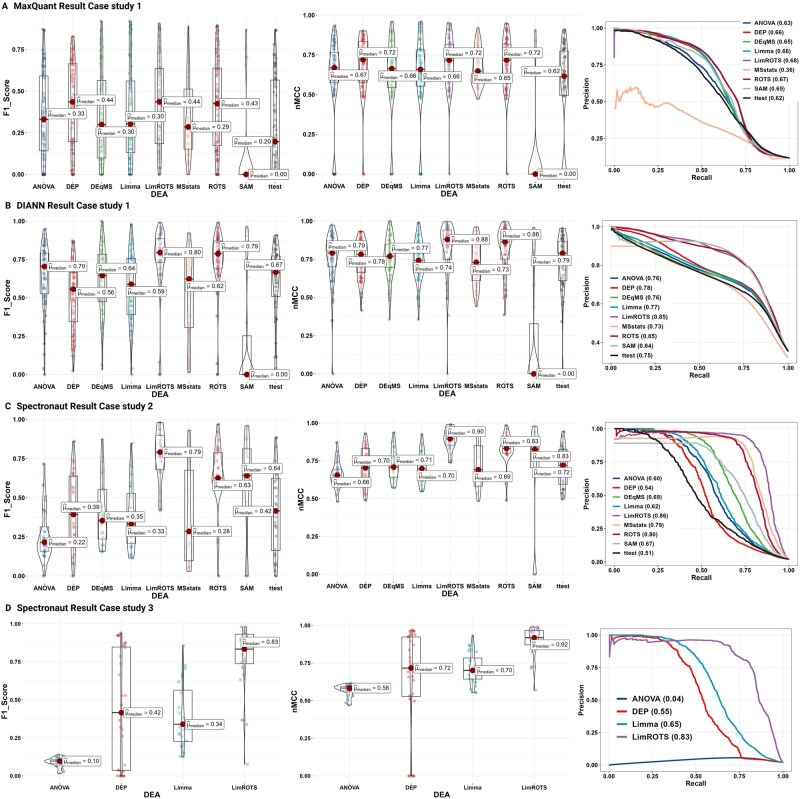
Benchmarking performance metrics; F1 score, nMCC, and PR-curve. (A) The benchmarking performance results (F1 score and nMCC) boxplots (with violin plot when it possible) for each method from case study 1 (Quantified by MaxQuant software), as well as the PR-curve with AUC scores annotated in the figure by the name of the methods. (B) The benchmarking performance results (F1 score and nMCC) boxplots (with violin plot when it possible) for each method from case study 1 (Quantified by DIA-NN software), as well as the PR-curve with AUC scores annotated in the figure by the name of the methods. (C) The benchmarking performance results (F1 score and nMCC) boxplots (with violin plot when it possible) for each method from case study 2 (Quantified by Spectronaut software), as well as the PR-curve with AUC scores annotated in the figure by the name of the methods. (D) The benchmarking performance results (F1 score and nMCC) boxplots (with violin plot when it possible) for LimROTS, limma, ANOVA, and DEP in case study 3 (Quantified by Spectronaut software), as well as the PR-curve with AUC scores annotated in the figure by the name of the methods.

In Case study 1, for the DDA dataset, when MaxQuant was used as the quantification software, DEP, LimROTS and ROTS outperformed other methods in terms of F1 score (0.44, 0.44, and 0.43, respectively) and nMCC (0.72, 0.72, 0.72, respectively) (Fig. 1A). Moreover, LimROTS and ROTS scored the highest in the PR-curve with 0.68 and 0.67 AUC, respectively (Fig. 1A). Similarly, when FragPipe was used as the quantification software, the three methods continued to be the top-performing methods overall (Fig. 1A, available as supplementary data at Bioinformatics online); however, performance across all tools was notably lower in terms of F1 score (Fig. S1A and Supplementary File 1, Table 1A and B, available as supplementary data at Bioinformatics online).

For the DIA datasets in case study 1, when DIA-NN was used as quantification software. With a significance threshold of 5% FDR, LimROTS and ROTS outperformed other methods in terms of nMCC (median values: 0.88 and 0.86, respectively), F1 score (median values: 0.80 and 0.78, respectively) (Fig. 1B), and pAUC 5% (median values: 0.94 and 0.93, respectively) (Supplementary File 1, Table 1C and D, available as supplementary data at Bioinformatics online). Furthermore, LimROTS, ROTS, DEP, DEqMS, and SAM had the highest AUC score in the PR curve with 0.85, 0.85, 0.78, 0.76 and 0.84 respectively (Fig. 1B). A similar trend was observed when Spectronaut was used as quantification software (Fig. 1B, available as supplementary data at Bioinformatics online). The results for all evaluation metrics across datasets and tools used in this study are available in Supplementary File 1, Table 1C and D, available as supplementary data at Bioinformatics online.

### 3.2 Case study 2: Dataset with two different DIA settings simulating a batch effect

In Case study 2, using Spectronaut as quantitation software, LimROTS clearly outperformed all the other methods with a median F1 score of 0.79 and 0.90 nMCC (Fig. 1C). Also, as displayed in the PR-curve plot, LimROTS showed a high PR ratio with AUC 0.86 (Fig. 1C).

Even when DIA-NN was used for quantification, LimROTS continued to exhibit the highest performance, with F1 score (median 0.87, with ROTS second at 0.82), nMCC (median 0.93, with ROTS second at 0.91), compared to other methods (Fig. 1C, available as supplementary data at Bioinformatics online). The results for all evaluation metrics across datasets and tools used in this case study are available in Supplementary File 1, Table 1A and B, available as supplementary data at Bioinformatics online.

### 3.3 Case studies 3 and 4: Evaluating LimROTS, ANOVA, DEP and limma in more complex semi-synthetic datasets

In these case studies, we excluded SAM, ROTS, and t-test, as these methods are not specifically designed to account for covariates in an experiment, which is crucial for obtaining accurate results, as demonstrated in Case Study 2. DEqMS and MSstats demonstrated a good overall performance in case studies 1 and 2, as they can manage covariates. However, in Case Studies 3 and 4, a synthetic batch effect was introduced at the protein expression level rather than at the peptide level. This makes the comparison incompatible with DEqMS and MSstats, which requires peptide-level input. As such, DEqMS and MSstats were excluded from these cases.

Therefore, LimROTS was compared against limma, ANOVA and DEP in this analysis. In case 3, using Spectronaut for the quantification, LimROTS demonstrated significantly higher performance across all evaluation metrics. Specifically, LimROTS achieved values of 0.83 (F1 score), 0.92 (nMCC), 0.96 (pAUC 5%), 0.95 (G-mean), while limma achieved values of 0.34, 0.70, 0.84, and 0.88, respectively. DEP scored 0.42, 0.72, 0.74, and 0.56, respectively. ANOVA scored 0.10, 0.58, 0, and 0.76, respectively as shown in Fig. 1D. Similarly, using DIA-NN as the quantification software, LimROTS outperformed limma and DEP in nMCC (0.93 compared to 0.70 limma, 0.58 ANOVA and 0.88 DEB) and F1 score (0.84 compared to 0.33 limma, 0.09 ANOVA, and 0.75 DEB) (Fig. 1D, available as supplementary data at Bioinformatics online and Supplementary File 1, Table 3A and B, available as supplementary data at Bioinformatics online). Furthermore, LimROTS outperformed both in the PR-curve with 0.83 AUC compared to 0.65 limma, 0.04 ANOVA, and 0.55, with Spectronaut expression matrix. Comparable results were obtained when DIA-NN was used in place of Spectronaut.

**Figure 2.**
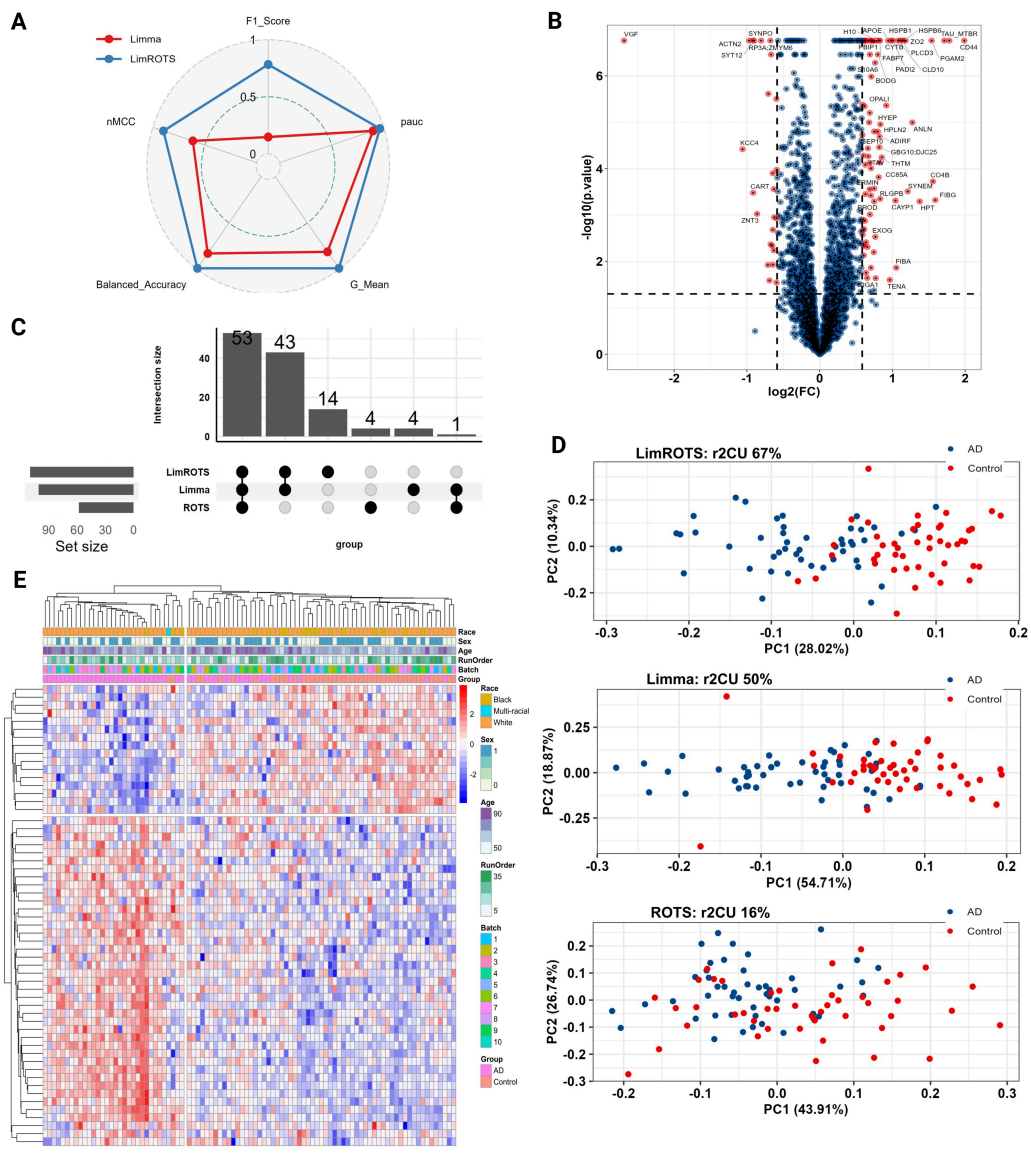
Benchmarking performance for case study 4 and UPenn cohort results. (A) A spider plot illustrates the performance differences between LimROTS (blue) and limma (red) in case study 4, using five metrics: F1 score, nMCC, pAUC5%, G-mean, and balanced accuracy. (B) The volcano plot shows the DEPs (*P*-value < 0.05 and FC ≥ log2(1.5)) represented by red dots from LimROTS, annotated with gene symbols. (C) UpSet displays the overlap DEPs between the three methods (LimROTS, ROTS, and limma), defined by the cutoffs (FDR < 0.05 and FC ≥ log2(1.5)). (D) PCA plots for non-overlapped DEPs identified using each method, from LimROTS, limma, and ROTS. Red and blue dots represent the Control and AD groups, respectively. Above each PCA, the Cragg and Uhler’s pseudo r-squared (r2CU) from fitting the PC1 with GLM, is displayed. (E) Heatmap with hierarchical clustering to the samples and LimROTS DEPs. Samples were annotated with Race, Sex, Age, Ms Run Order, and Digestion batches. This heatmap shows that no batch effects are present and that the clustering is clearly driven by disease state (AD vs Control).

Based on these results, and as DEP statistical framework uses limma, case study 4 will include only LimROTS and limma. In Case 4, as shown in Fig. 2A and Supplementary File 1, Table 4, available as supplementary data at Bioinformatics online, LimROTS notably outperformed limma across all evaluated metrics. Specifically, LimROTS and limma achieved the following scores: F1 score (0.86 compared to 0.14), nMCC (0.93 compared to 0.61), pAUC 5% (0.98 compared to 0.90), G-mean (0.99 compared to 0.81), and balanced accuracy (0.99 compared to 0.83).

### 3.4 UPenn case study: Performance of DEA tools using real-world clinical proteomics data

In order to evaluate LimROTS performance with real-world clinical data, we used the UPenn cohort (Johnson *et al.* 2020). This cohort contains post-mortem brain samples from a range of neurodegenerative diseases. We used only the Alzheimer’s disease (AD) cases compared to healthy controls. After applying limma, ROTS, and LimROTS, proteins with less than 0.05 adjusted *P*-value and log2 1.5-fold change were identified as differentially expressed proteins (DEPs) (Supplementary File 2, Tables 1–3, available as supplementary data at Bioinformatics online, and Fig. 2B). The UpSet plot (Fig. 2C) presents the number of DEPs that overlap and the number that are unique, comparing the three methods. A PCA using protein expression matrix was used to assess the degree to which the uniquely identified significant proteins from each testing method represented biological diversity between the AD and Control groups (Fig. 2D). We then fitted a generalized linear model (GLM) to each PC1 with diagnostic (binary) status as a response variable (represented as Diagnosis ∼ PC1), then Cragg and Uhler’s pseudo r-squared was computed (r2CU).

Additionally, the prediction probabilities from GLM were then utilized to compute the receiver operating characteristic (ROC), with diagnostic groups (Control and AD) serving as the response variable and the GLM prediction probabilities as the predictor variable for each sample. LimROTS achieved the highest r2CU (67%) and 0.93 area under the ROC (AUC), followed by limma with r2CU equal to 50% and 0.87 AUC, lastly ROTS achieved only 16% and AUC equal to 0.7 (Fig. 1 and Fig. 2, available as supplementary data at Bioinformatics online). Furthermore, we fitted another GLM directly to the significant protein list from each tool (without PCA), as direct modeling using the protein list could retain more biological signals. From this model, LimROTS scored the highest (r^2^CU = 0.79, AUC = 0.96), followed by Limma (r^2^CU = 0.52, AUC = 0.88) and finally ROTS (r^2^CU = 0.40, AUC = 0.80).

Next, the DEPs from LimROTS (Fig. 2B) were used for enrichment analysis using two databases; DisGeNET and Jensen DISEASES, using Enrichr server (Xie *et al.* 2021). With significant cutoff less than 0.05 adjusted *P*-value (Fig. 2, available as supplementary data at Bioinformatics online). Moreover, a heat-map with a hierarchical clustering was generated using the DEPs from LimROTS, with annotation for race, sex, age, MS run order, and digestion batches. The clustering analysis showed that samples mostly grouped by the diagnostic annotation (Control and AD), with no apparent impact from other annotated variables (Fig. 2E).

## 4 Discussion

In Case study 1, the findings suggest that LimROTS and ROTS offer superior overall performance compared to alternative methods (e.g. MSstats, DEP, DEqMS, limma, t-test, ANOVA, and SAM) when DIA-NN or Spectronaut is used as the quantification software using a significance threshold of 5% FDR. The high nMCC values, which reflect the overall classification performance, indicate that both LimROTS and ROTS accurately detect most true positive proteins while effectively distinguishing true negatives. The F1 score, a metric that balances precision and recall, was also higher for LimROTS and ROTS, further supporting their superior capability in detecting significant proteins while minimizing false positives and false negatives. Additionally, both methods showed strong performance in terms of PR (precision-recall curves) AUC, highlighting their ability to identify the positive proteins with higher precision across a range of recall values. Furthermore, for DDA-based analysis, particularly when using MaxQuant for quantification, LimROTS and ROTS continued to outperform other methods (with the addition of DEP). These results establish LimROTS as promising candidates for DEA in proteomics DDA and DIA workflows.

The findings from Case Study 2, where the experimental design complexity increased by combining the same set of samples analyzed using both narrow and wide DIA settings, indicate that LimROTS showed significantly higher overall performance compared to all other tools. The highest F1 score, 0.76, was achieved by LimROTS, indicating its superior ability to identify true positives while minimizing both false positives and false negatives. In contrast, the second-highest score of 0.66 was recorded by ROTS, which further suggests that LimROTS is more effective, even in the presence of covariates. Furthermore, LimROTS achieved the highest scores for both nMCC and PR AUC, indicating better discriminative power and stronger overall performance in distinguishing between true differential and non-differential proteins. These results can be attributed to the additional advantages LimROTS offers over ROTS, as it flexibly integrates covariates in the linear model, along with its advantage over limma and other methods by optimizing the rank statistics of the proteins.

Comparing LimROTS, ANOVA, DEP, and limma in case studies 3 and LimROTS with limma in case study 4, demonstrates that LimROTS significantly outperforms other methods, in balancing precision and recall when using either Spectronaut or DIA-NN. LimROTS effectively identifies true positives while minimizing false positives and false negatives, as indicated by its high F1 score. Additionally, the G-mean, which represents the balance between sensitivity (true positive rate) and specificity (true negative rate), was higher for LimROTS. Furthermore, the higher pAUC 5% score and PR AUC of LimROTS highlights its superior ability to rank the top 5% of the most DEPs with greater confidence in its top-ranking results. Moreover, the nMCC score of LimROTS exceeded the performance of limma, ANOVA, and DEP. Overall, LimROTS consistently outperformed other methods when using either Spectronaut or DIA-NN, which indicates that LimROTS is a more reliable and accurate tool for proteomics data. Case Study 4 particularly features a more complex experimental design than the other case studies, requiring a flexible tool to effectively model covariates, even in unbalanced settings. The results from this case show that LimROTS performs better at accurately identifying DEPs while maintaining a strong balance between sensitivity and specificity.

Finally, using the UPenn cohort, unique proteins that only identified as DEPs with LimROTS (14 proteins) demonstrated the greatest r2CU of 67% and with AUC-ROC 0.93, signifying that the DEPs are attributable to the biological disparities between the studied AD and control groups. In this analysis ROTS showed less significant proteins compared to LimROTS and limma (Fig. 2C), which affected its PCA and r2CU dramatically. This can be due to the batch correction method (Combat) used to pre-process the dataset before applying ROTS test, which is essential for the analysis. However, this batch correction step could inadvertently eliminate biological signals (Phua *et al.* 2022, Wang and Cao 2023, Hui *et al.* 2024). In contrast, LimROTS has the advantage of incorporating the batch information as a fixed variable in the model, accounting for it during the DEA. Furthermore, the heatmap illustrated that the DEPs from LimROTS are not due to technical bias. Rather, the samples clustered according to diagnostic groups (AD and Control) and not according to technical or unwanted biological variables such as age or sex.

In the UPenn cohort, fourteen unique DEPs were identified using LimROTS (Supplementary File 2, Table 4, available as supplementary data at Bioinformatics online). Among the upregulated proteins is chimerin-1, a GTPase-activating protein for p21-Rac. It was previously shown to be regulated at the transcriptome level in late-onset AD (Arzouni *et al.* 2020). Chimerin was shown to regulate microglial migration to amyloid-beta deposits in AD (Chen *et al.* 2022). PDLIM5, which was also elevated in AD samples, plays a role in neuronal development, synaptic assembly, and dendritic spine formation, and is identical to AD7c-NTP, an AD-related protein, and has been connected with psychiatric disorders and polygenic risk score for AD (Herrick *et al.* 2010, Miao *et al.* 2020). Ragulator complex protein 5 (LAMTOR5) was also shown to be upregulated when analysed with LimROTS. This protein activates mTORC1 which inhibits autophagy and may contribute to protein aggregation in AD (Ma *et al.* 2020). Proteins downregulated in AD included PITRM1. Loss-of-function in this protein correlates with mitochondrial dysfunction and neurodegeneration (Brunetti *et al.* 2021). These findings support earlier associations between these proteins and AD pathogenesis.

Normalization is essential in proteomics because it eliminates unwanted variation from sample handling and instrument drift, allowing datasets to be directly compared. Without proper normalization, biases inflate variance and obscure true biological signals. Advanced methods such as MaxLFQ (Cox *et al.* 2014) and directLFQ (Ammar *et al.* 2023) address these issues by correcting systematic errors and estimating protein intensities reliably, even in cases of missing values or varying peptide baselines. Simpler strategies, like median normalization, apply a single scaling factor across all proteins, which can fail in experiments with widespread protein changes or inconsistent data coverage. Throughout the study, we preprocessed every proteomics dataset with directLFQ (Ammar *et al.* 2023), then imputed missing values without applying any global normalization (no median or quantile normalization). We used the resulting matrices as input to all DEA methods. We followed this pipeline because it performed best overall across multiple experimental settings (Peng *et al.* 2024). Since directLFQ pulls together peptide intensities, normalizes at the protein level across samples, estimates protein abundance, and handles missing data, extra global normalization is often unnecessary. In contrast, approaches using spectral counts, “Top0” (using all available precursors), and “Top3” (using the three most intense precursors) generally benefit from careful global normalization. The only exception to these preprocessing steps in this study was the set of input parameters specified for MSstats. Because MSstats is designed with a fixed set of quantification, normalization, and imputation options, replacing these with new algorithms would not be straightforward, making direct comparisons to other tools imperfect. To keep things as comparable as possible, we omitted global normalization for MSstats as well and Tukey’s median polish was used as the summarization method.

## 5 Limitations and future perspective

While bootstrapping and the permutation techniques utilized in LimROTS are statistically robust, these techniques are computationally more expensive. We attempted to resolve this issue by incorporating parallel processing. This significantly reduced computational time, but at the cost of increased Random-access memory (RAM) utilization. In future versions, we intend to enhance the workflow to speed up the analysis while minimizing the requirement for substantial computational resources. Initially, we will convert the optimization step into a C++ code and integrate it into R using Rcpp package, this could be expected to increase the speed of this step dramatically. Additionally, the bootstrapping and permutation steps could also be parallelized for simultaneous execution, however, prior to this, we should optimize the code for enhanced speed independent of the parallelization. Furthermore, incorporating additional functionality for data preprocessing and visualization. Our benchmarks cover software versions available on October 30, 2024 (i.e., Bioconductor 3.20). While research software is a living concept, users should treat experimental features that lack independent verification with caution.

## 6 Conclusion

LimROTS consistently outperformed other DEA statistical methods such as limma, ROTS, MSstats, DEqMS, DEP and SAM, in diverse experimental contexts. It exhibited exceptional performance across essential evaluation measures, irrespective of the quantification approach employed. LimROTS demonstrated efficacy in managing intricate experimental designs, optimizing recall and precision, and addressing biases and imbalanced batches more effectively than alternative techniques. Moreover, it yielded biologically significant outcomes in clinical proteomics data, hence enhancing its credibility. In this study we focused on proteomics data; however, LimROTS may have wider relevance in other omics domains, including transcriptomics and metabolomics, pending additional validation. Ultimately, LimROTS proved to be a more precise, dependable, and adaptable method for DEA in high-dimensional omics research.

## Supplementary Material

btaf570_Supplementary_Data

## Data Availability

The 21 Datasets used in case study 1,2, and 3 are available under the OpDEA resource at http://www.ai4pro.tech:3838, https://zenodo.org/records/10953347, and https://zenodo.org/records/10482353 adapted from (Peng *et al.* 2024), the ProteomeXchange repository IDs and the original study for each dataset are available in table 1. The dataset used in case study 4 is available at ProteomeXchange repository with ID number: PXD026600 (Gotti *et al.* 2022, 2021). For the UPenn cohort case study, the dataset was obtained from the AD Knowledge Portal (https://adknowledgeportal.org), specifically from dataset DOI: https://doi.org/10.7303/syn20933797. To facilitate reproducibility, we deposited all datasets and the code required to run the analyses presented in this study in Zenodo: https://doi.org/10.5281/zenodo.17102211

## Code availability

LimROTS has been implemented as an R/Bioconductor package, available at https://doi.org/doi:10.18129/B9.bioc.LimROTS. Additionally, the code used in this study is available in the GitHub repository: https://github.com/AliYoussef96/LimROTSmanuscript. The package supports the R/Bioconductor SummarizedExperiment data structure (Huber *et al.* 2015), which enhances interoperability with the vast array of other Bioconductor methods.
