# Supplementary material for: LimROTS: a hybrid method integrating empirical Bayes and reproducibility-optimized statistics for robust differential expression analysis
Source: Bioinformatics. 2025 Oct 11;41(12):btaf570. doi: 10.1093/bioinformatics/btaf570 (PMC12674742; doi:10.1093/bioinformatics/btaf570)
Supplement: btaf570_Supplementary_Data [file btaf570_supplementary_data.zip › Supplementary.docx]

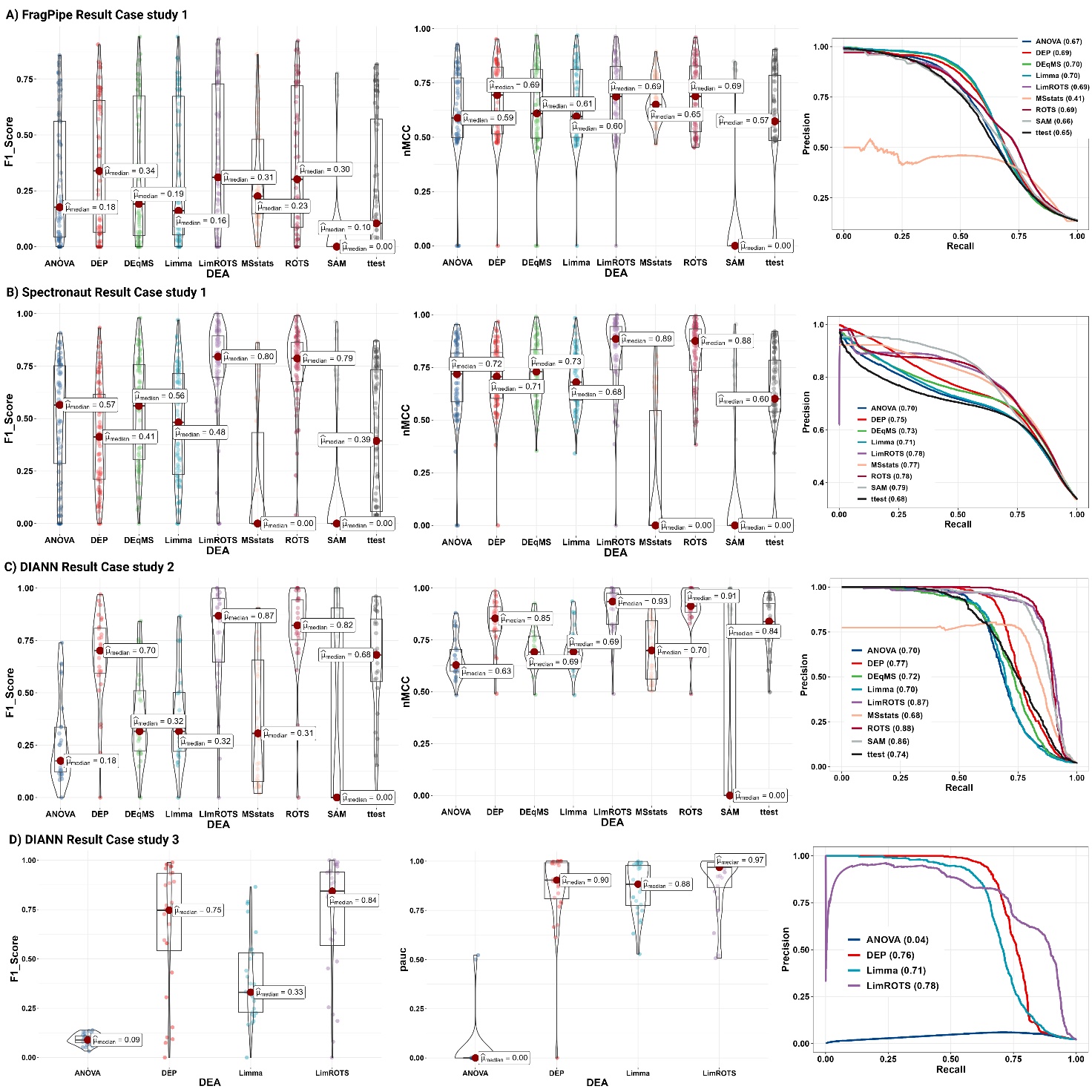


**Figure S1:** Benchmarking Performance Metrics; F1 score, nMCC, and PR-curve. A: The benchmarking performance results show F1 score and nMCC (as boxplots, for each method from case study 1 (Quantified by FragPipe software), as well as the PR-curve with AUC scores annotated in the figure with the method’s name. B: The benchmarking performance results show F1 score and nMCC as boxplots (with violin plot when possible) for each method from case study 1 (Quantified by Spectronaut software), as well as the precision-recall (PR)-curve with AUC scores annotated in the figure with the method’s name. C: The benchmarking performance results show (F1 score and nMCC) boxplots (with violin plot when possible) for each method from case study 2 (Quantified by DIA-NN software), as well as the PR-curve with AUC scores annotated in the figure by the name of the methods. D: The benchmarking performance results (F1 score and nMCC) boxplots for LimROTS, limma, ANOVA and DEP in case study 3 (Quantified by DIA-NN software), as well as the PR-curve with AUC scores annotated in the figure by the name of the methods.


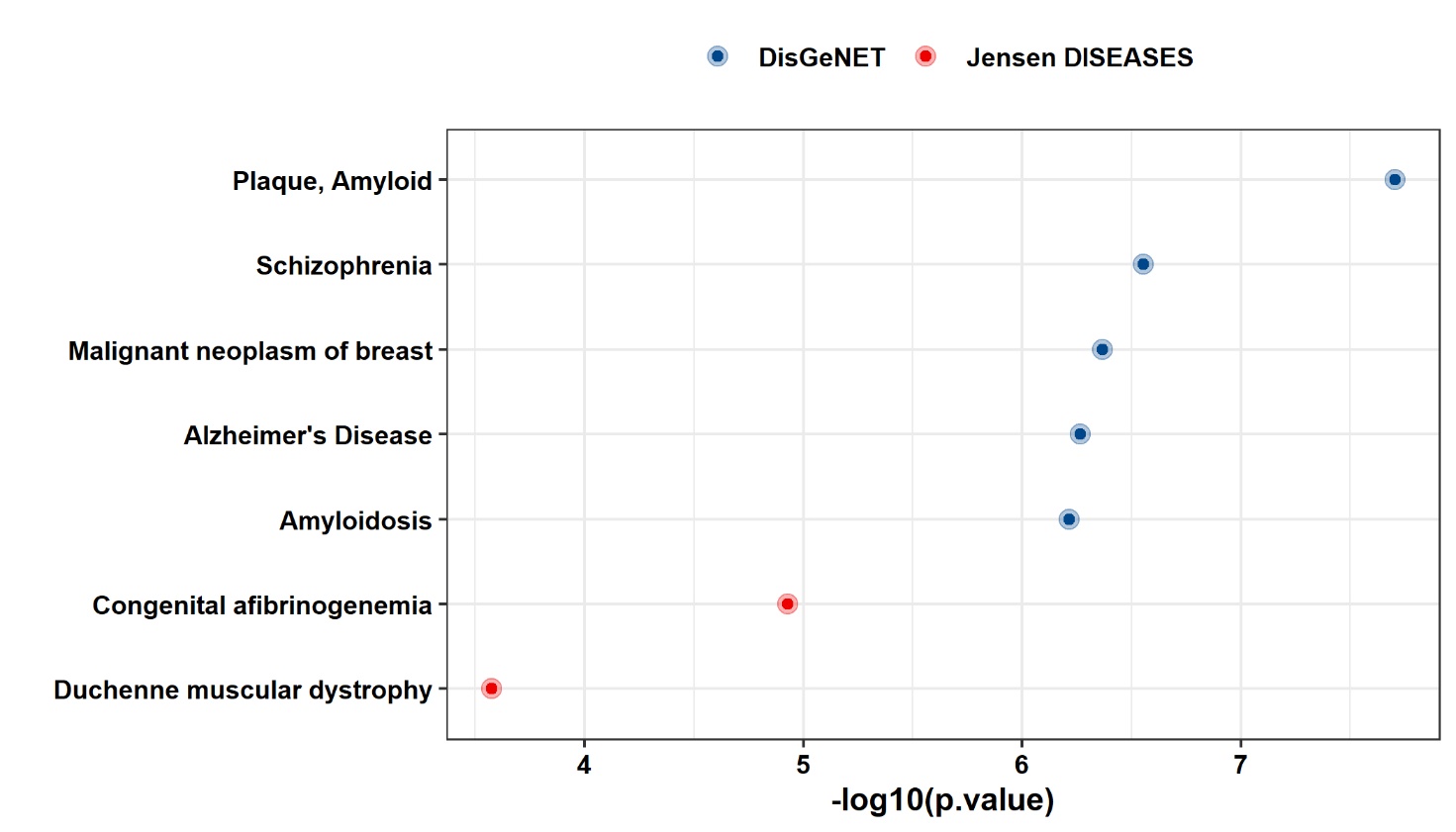


**Figure S2:** Enrichment analysis for significant proteins identified using LimROTS.
Enrichment analysis using two databases; DisGeNET and Jensen DISEASES, using Enrichr server. With significant cutoff less than 0.05 adjusted p-value. For significant proteins identified using LimROTS.


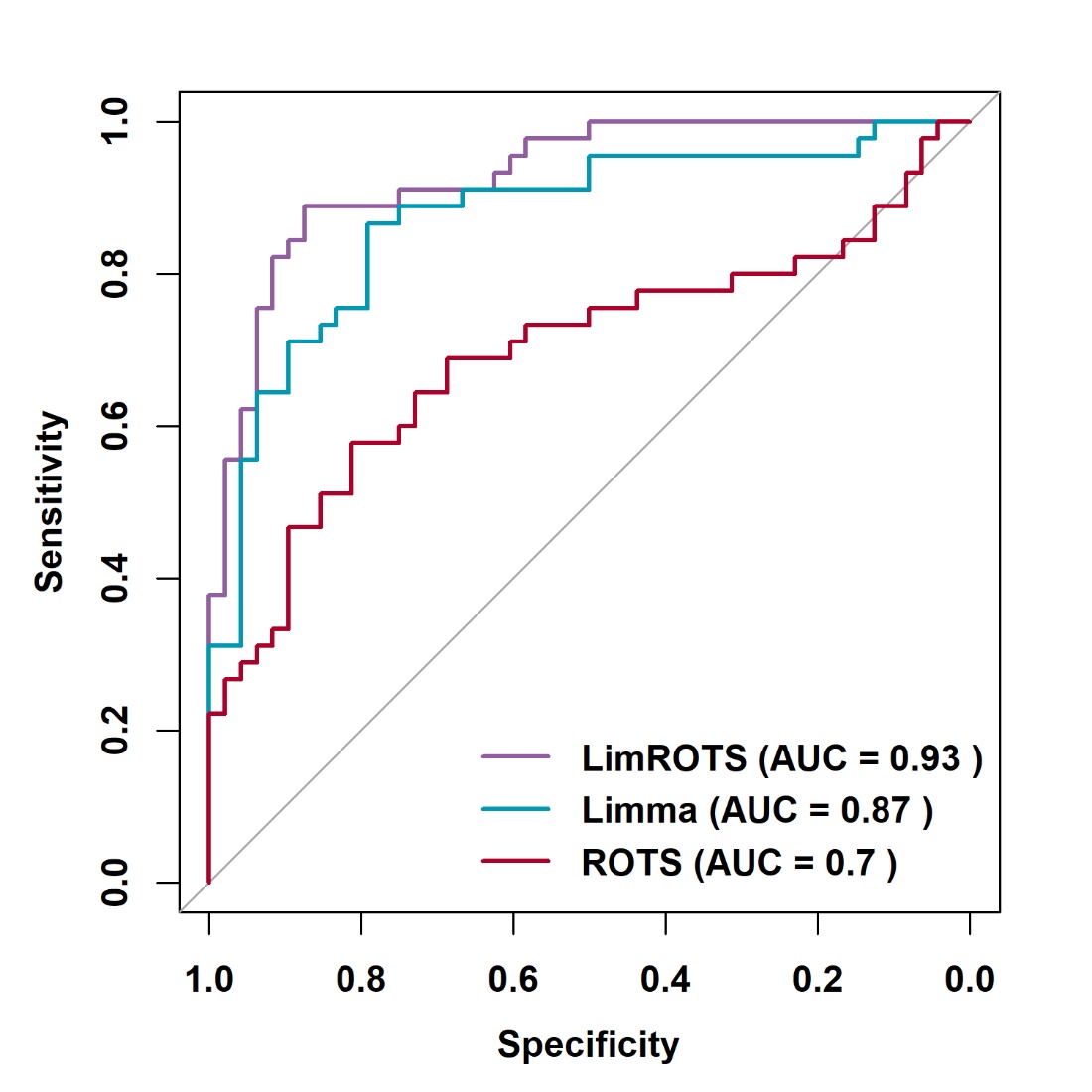


**Figure S3:** Receiver operating characteristic (ROC), with diagnostic groups (Control and AD) as the response variable and the GLM prediction probabilities as the predictor variable for each sample. For each method (LimROTS, limma, and ROTS) A PCA was generated using the distinct collection of significant proteins identified by each method. We subsequently employed a generalized linear model (GLM) for each PC1, using diagnostic (binary) status as the response variable (represented as Diagnosis ~ PC1 AD Vs. Control).  The GLM prediction probabilities are utilized to compute the ROC, with diagnostic groups (Control and AD) serving as the response variable and the GLM prediction probabilities as the predictor variable for each sample. Additionally, the AUC was annotated along with the method name.

**Supplementary file 1:** The supplementary file includes tables that display the performance metrics assessed across all case studies for every method. Table S1: Findings from Case Study 1, Table S2: Findings from Case Study 2, Table S3: Findings from Case Study 3, and Table S4: Findings from Case Study 4.

**Supplementary file 2:** The supplementary file has three tables: Table S1 presents the DEA statistical results from LimROTS (p-value, q-value, and log2 fold change), Table S2 offers equivalent data from ROTS, and Table S3 includes results from limma. Table S4 contains the 14 unique LimROTS DEPs.
