# Supplementary figures and images for: LimROTS: a hybrid method integrating empirical Bayes and reproducibility-optimized statistics for robust differential expression analysis
Source: Bioinformatics. 2025 Oct 11;41(12):btaf570. doi: 10.1093/bioinformatics/btaf570 (PMC12674742; doi:10.1093/bioinformatics/btaf570)

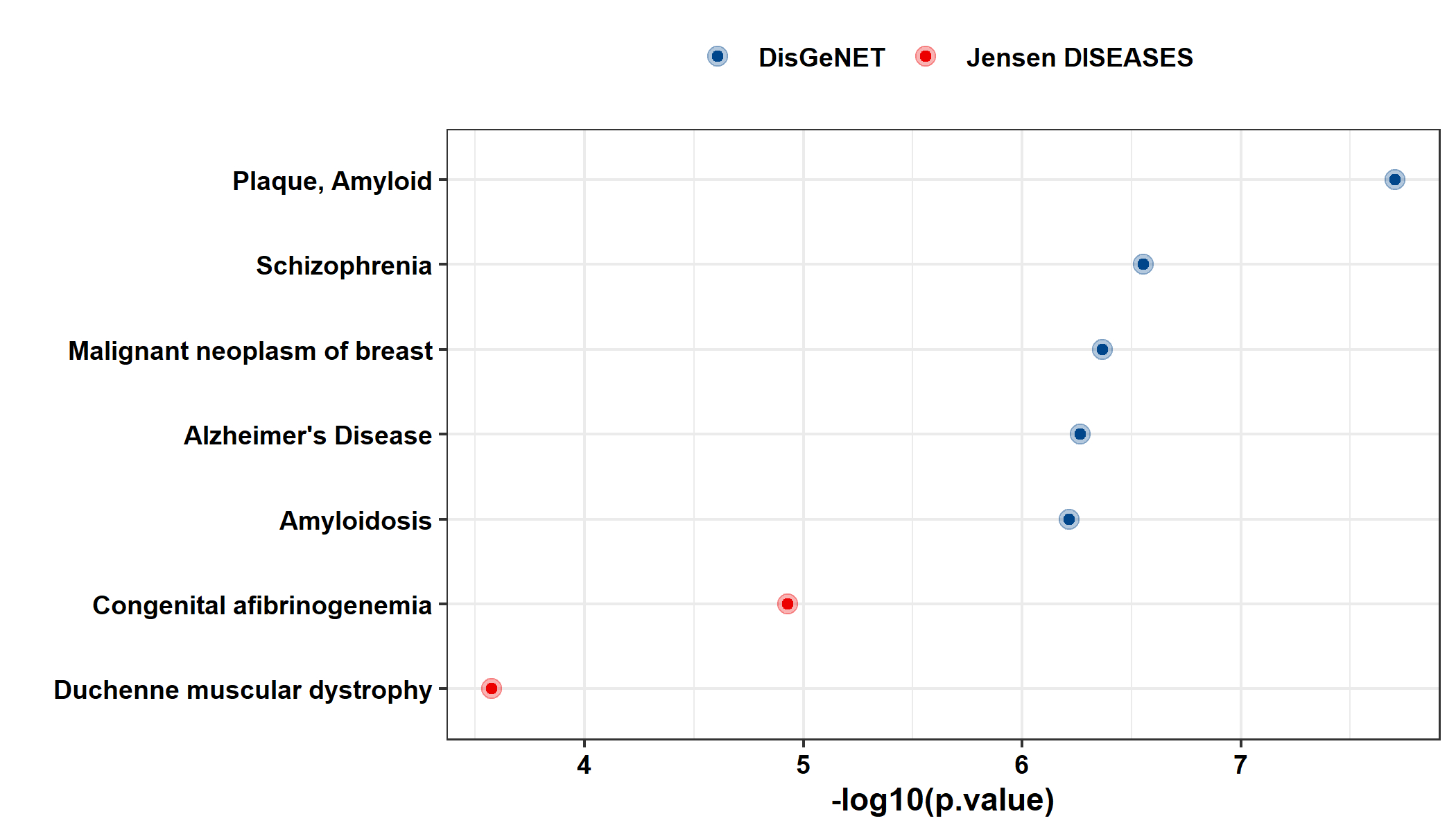

Supplement: btaf570_Supplementary_Data [file btaf570_supplementary_data.zip › Figure 2S.jpg]

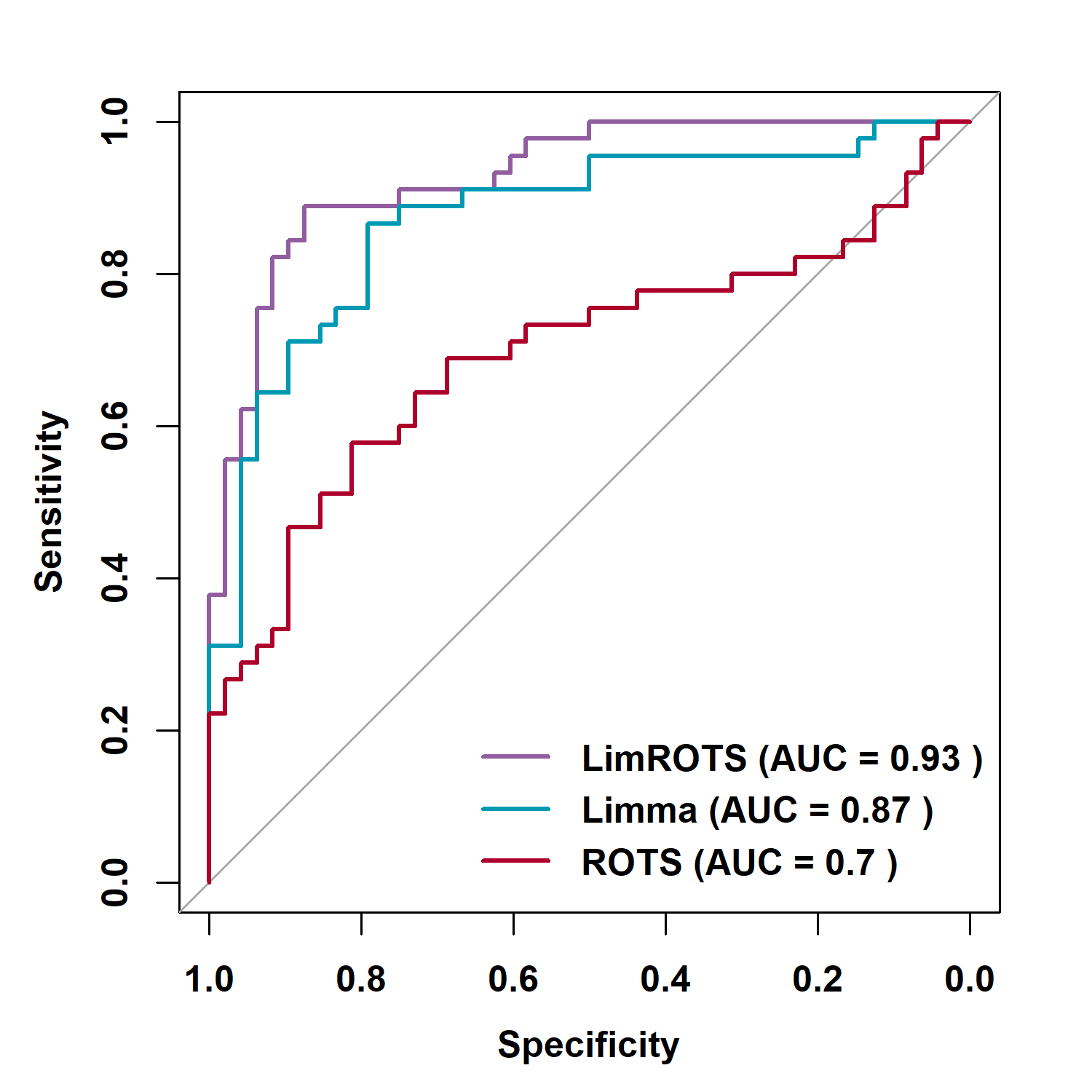

Supplement: btaf570_Supplementary_Data [file btaf570_supplementary_data.zip › Figure 3S.jpg]

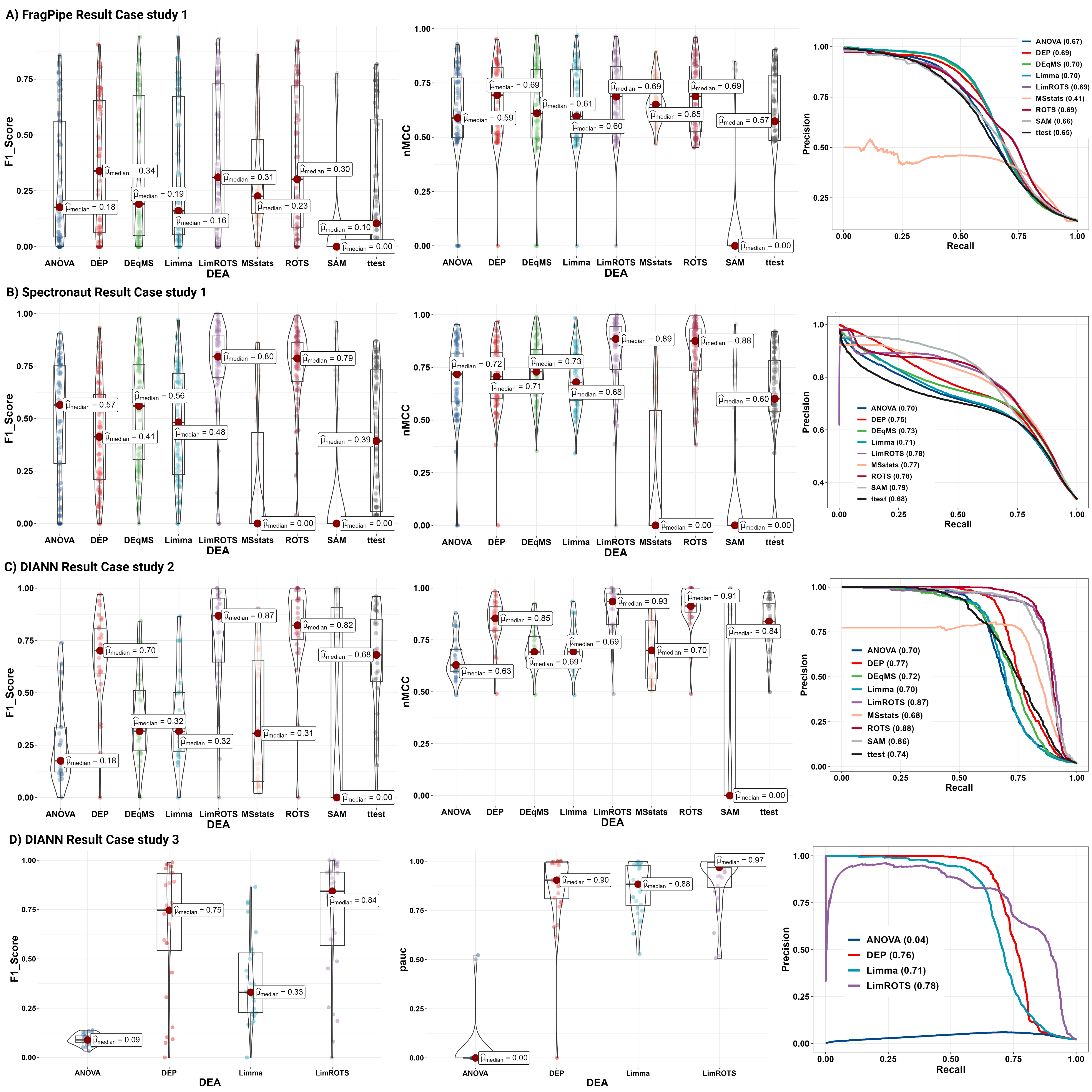

Supplement: btaf570_Supplementary_Data [file btaf570_supplementary_data.zip › Figure S1.jpeg]
